# Mutation rate, selection, and epistasis inferred from RNA virus haplotypes via neural posterior estimation

**DOI:** 10.1093/ve/vead033

**Published:** 2023-05-20

**Authors:** Itamar Caspi, Moran Meir, Nadav Ben Nun, Reem Abu Rass, Uri Yakhini, Adi Stern, Yoav Ram

**Affiliations:** Shmunis School of Biomedicine and Cancer Research, Faculty of Life Sciences, Tel Aviv University, P.O. Box 39040, Tel Aviv 6997801, Israel; Shmunis School of Biomedicine and Cancer Research, Faculty of Life Sciences, Tel Aviv University, P.O. Box 39040, Tel Aviv 6997801, Israel; Edmond J. Safra Center for Bioinformatics, Tel Aviv University, P.O. Box 39040, Tel Aviv 6997801, Israel; School of Zoology, Faculty of Life Sciences, Tel Aviv University, P.O. Box 39040, Tel Aviv 6997801, Israel; Shmunis School of Biomedicine and Cancer Research, Faculty of Life Sciences, Tel Aviv University, P.O. Box 39040, Tel Aviv 6997801, Israel; Shmunis School of Biomedicine and Cancer Research, Faculty of Life Sciences, Tel Aviv University, P.O. Box 39040, Tel Aviv 6997801, Israel; Edmond J. Safra Center for Bioinformatics, Tel Aviv University, P.O. Box 39040, Tel Aviv 6997801, Israel; Shmunis School of Biomedicine and Cancer Research, Faculty of Life Sciences, Tel Aviv University, P.O. Box 39040, Tel Aviv 6997801, Israel; Edmond J. Safra Center for Bioinformatics, Tel Aviv University, P.O. Box 39040, Tel Aviv 6997801, Israel; Edmond J. Safra Center for Bioinformatics, Tel Aviv University, P.O. Box 39040, Tel Aviv 6997801, Israel; School of Zoology, Faculty of Life Sciences, Tel Aviv University, P.O. Box 39040, Tel Aviv 6997801, Israel

**Keywords:** mutation rate, neural networks, likelihood free inference, ms2, experimental evolution

## Abstract

RNA viruses are particularly notorious for their high levels of genetic diversity, which is generated through the forces of mutation and natural selection. However, disentangling these two forces is a considerable challenge, and this may lead to widely divergent estimates of viral mutation rates, as well as difficulties in inferring the fitness effects of mutations. Here, we develop, test, and apply an approach aimed at inferring the mutation rate and key parameters that govern natural selection, from haplotype sequences covering full-length genomes of an evolving virus population. Our approach employs *neural posterior estimation*, a computational technique that applies simulation-based inference with neural networks to jointly infer multiple model parameters. We first tested our approach on synthetic data simulated using different mutation rates and selection parameters while accounting for sequencing errors. Reassuringly, the inferred parameter estimates were accurate and unbiased. We then applied our approach to haplotype sequencing data from a serial passaging experiment with the MS2 bacteriophage, a virus that parasites *Escherichia coli*. We estimated that the mutation rate of this phage is around 0.2 mutations per genome per replication cycle (95% highest density interval: 0.051–0.56). We validated this finding with two different approaches based on single-locus models that gave similar estimates but with much broader posterior distributions. Furthermore, we found evidence for reciprocal sign epistasis between four strongly beneficial mutations that all reside in an RNA stem loop that controls the expression of the viral lysis protein, responsible for lysing host cells and viral egress. We surmise that there is a fine balance between over- and underexpression of lysis that leads to this pattern of epistasis. To recap, we have developed an approach for joint inference of the mutation rate and selection parameters from full haplotype data with sequencing errors and used it to reveal features governing MS2 evolution.

## Introduction

Mutations are one of the primary sources of genetic heterogeneity in viruses and can be seen as the fuel of evolution. The mutation rate is defined as the number of new mutations in a genome over a unit of time, usually one generation. Viruses are notorious for their extremely high mutation rates (Sanjuán et al. 2010; Zanini et al. 2017; Duffy 2018). Therefore, the viral mutation rate is a key parameter of virus evolution that, together with selection, determines the extent to which genetic diversity is created in a population of viruses.

Current methods for measuring the mutation rate often involve genomic sequencing of viral populations across time. Experimental evolution of viral populations is a powerful way to track viral mutations: in a controlled laboratory serial passaging experiment, a viral population is allowed to replicate for several generations, and deep sequencing is used to measure the dynamics of mutant allele frequencies. The high viral mutation rate will lead to many new mutations being constantly introduced; selection will eliminate deleterious mutations and will lead to an increase in the frequency of beneficial mutations that promote viral fitness. The challenge is then, when observing mutations and their frequencies, to separate between the effects of mutation and natural selection, which constantly affects allele frequencies even during a single round of replication (Peck, Lauring, and Sullivan 2018). One way of overcoming this challenge is to focus on specific mutations with known fitness effects. For example, one may focus on lethal mutations and measure their frequency because, under the mutation–selection balance, the frequency of lethal mutations should be equal to the mutation rate (Cuevas et al. 2009; Acevedo, Brodsky, and Andino 2014). Focusing on lethal mutations leads to several subsequent challenges: first, defining mutations as lethal is not always straightforward; second, those lethal mutations that can be defined may be a small subset of all loci; finally, lethal mutations may not be easily distinguished from sequencing errors (but see Acevedo, Brodsky, and Andino 2014).

Another approach is to focus on neutral mutations. In theory, if evolution begins with a completely homogeneous population, neutral mutations are expected to accumulate at the rate of mutation. The challenge, then, is to specify which mutations are neutral. In some studies, synonymous mutations are assumed to be neutral (Tromas and Elena 2010; Stern et al. 2017; Zanini et al. 2017; Zinger et al. 2019). However, there is growing evidence that many synonymous mutations are not neutral, and this may be particularly exacerbated in viruses with small, dense genomes where genomic regions can have overlapping functions (Cuevas, Domingo-Calap, and Sanjuán 2012; Mayrose et al. 2013; Zanini and Neher 2013). An additional complication is that both the lethal mutation and neutral mutation approaches are based on single-locus models, neglecting multi-locus effects such as background selection, selective sweeps (Feder, Pennings, and Petrov 2021), and epistatic interactions.

Here, we focus on MS2 bacteriophage, +ssRNA virus from the Leviviridae family that parasites *Escherichia coli*, which is a widely studied model virus. Nevertheless, the mutation rate of MS2 has yet to be estimated. Mostly, estimates of mutation rates of Qβ, a close relative of MS2, are assumed to apply to MS2. These estimates vary widely, ranging from 0.08 (Garcia-Villada, Drake, and Hughes 2012) to 0.6 (Bradwell et al. 2013) to 6.5 (Batschelet, Domingo, and Weissmann 1976; Domingo, Flavell, and Weissmann 1976; Drake 1993) mutations per genome per replication cycle. This variation suggests that previous estimation approaches, based on only a handful of genomic loci, are not accurate enough (Garcia-Villada, Drake, and Hughes 2012). Importantly, MS2 has a particularly short genome of about 3,500 bases, allowing us to obtain reads that cover the entire genome.

Here, we develop and test an approach to jointly infer both the point mutation rate and selection parameters from sequencing data of MS2. Our approach relies on key methodological novelties: (1) long-read sequencing that covers the full length of the viral genome (i.e. haplotypes) (Callahan et al. 2021), (2) a multi-locus evolutionary model that captures the multitude of segregating haplotypes in the population, and (3) deep artificial neural networks and simulation-based inference that allow efficient and high-dimensional inference of model parameters (Avecilla et al. 2022). We applied this approach to infer the MS2 mutation rate and selection parameters from long-read haplotype sequences sampled from populations evolving in the laboratory.

## Methods

### Evolutionary experiment

Clonal MS2 stock was propagated from a single plaque to ensure that the experiment began with a phage population as genetically homogeneous as possible. Our experimental design was similar to the one described previously (Meir et al. 2020) with some changes: we performed ten serial passages at 37°C with three biological replicates (lines A–C). The serial passaging protocol was designed to allow tight population size control, to limit host–phage coevolution (naive *E. coli* c-3000 hosts were provided for each passage), and to limit coinfection and ecological interactions of phages within the cell, as described later.

We measured the length of the MS2 replication cycle by performing a one-step growth curve experiment as described below (Meir et al. 2020). After 120 minutes, we reached the maximal PFU of viruses after infection, and thus, passages were arrested after 120 minutes (Fig. S12).

The serial passages were performed at a multiplicity of infection (MOI) of 0.1 as follows: 10 ml cultures of naive *E. coli* c-3000 were grown to an optical density (OD) of OD_600_ = 0.4 (corresponding to a density of about 2 · 10^7^ cells/ml). Each passage was infected with 0.2 ml of 10^8^ phages from the previous passage, thus keeping an MOI of 0.1 PFU per cell (*N *= 2 · 10^7^ PFU). The cultures were grown for 120 minutes with shaking of 200 rpm, and *E. coli* cells were then removed by centrifugation. The supernatant was subjected to filtration with a 0.22-μm filter to remove any remaining residues. The new phage stock was then stored at 4°C. Aliquots of these phage stocks were used for measuring the concentration of phages by plaque assay (as described later), infecting the next serial passage, isolating RNA for whole-genome deep sequencing (as described later), and maintaining a frozen stock of the evolving lines in 15 per cent glycerol at −80°C. RNA was isolated using the QIAamp® viral RNA Mini kit (QIAGEN) according to the manufacturer’s instructions.

### Plaque assay

A plaque assay was performed at the end of each passage to determine the MS2 phage titer (Meir et al. 2020). Briefly, *E. coli* c-3000 was grown to mid-logarithmic phase (OD_600_ of 0.5) in rich growing medium Luria-Bertani (LB) at 37°C with shaking at 200 rpm. Serial dilutions of the MS2 samples were prepared in NaCl 0.85 per cent to reduce the phage concentration to less than 200 PFU/ml, which could be counted easily on a Petri dish with the naked eye. We added into each test tube 5 ml of soft agar (70 per cent) with 1 ml of *E. coli*. Then, 0.1 ml of each phage sample was added, and all of the tube content was emptied onto solid base agar in standard Petri dishes and allowed to harden. The plates were incubated overnight at 37°C.

### One-step growth curve

A culture of *E. coli* c-3000 was grown in LB medium at 37°C to an OD_600_ of 0.5 and then infected with wildtype MS2 at MOI = 0.1. At times 0, 30, 45, 60, 75, 90, 105, 120, 150, and 180 minutes post-infection, 1 ml was collected, centrifuged (1 minute at 13,000 rpm at room temperature), and the supernatant was filtered with a 0.22-μm filter to remove any remaining residues. Next, a plaque assay was performed to determine the concentration of the MS2 phage. A one-step growth curve was generated using the R *drc* package to fit to a log-logistic curve.

### Loop Genomics library construction, sequencing, and processing

RNA from passages 3, 7, and 10 from all three lines was sent to Loop Genomics (San Jose, CA, USA) and sequenced using the LoopSeq RNA preparation kit and its protocol (information available at loopgenomics.com). Loop Genomics’ synthetic long-read approach enabled full-length sequencing of the MS2 RNA genome with a low error rate of 5 · 10^−5^ errors per base (Callahan et al. 2021). In short, the LoopSeq method uses unique molecular barcode-labeling technology in which each barcode is distributed across a single genome followed by fragmentation of the genome into shorter fragments. The labeled fragmented genomes are then sequenced by existing standard short-read sequencing approaches on the Illumina sequencing platforms. The short-read raw data were uploaded to the Loop Genomics’ unique analytic pipeline. The pipeline was used for low-quality base trimming, unique sample barcode demultiplexing, and synthetic long-read reconstruction. The synthetic long-read reconstruction is a process that enables the de novo assembly to the full-length genomes after rearranging the short reads tagged with the same unique barcode.

### Data analysis

We ran BLAST (Altschul et al. 1990; McGinnis and Madden 2004) to align the long reads obtained from LoopSeq against the MS2 reference sequence (GenBank ID V00642.1, with some small differences noted in Meir et al. 2020) with the following parameters: --evalue 1e-07 --perc_identity 0.85 --task blastn --num_alignments 1000000 --dust no --soft_masking F. To ensure that we obtained only reads that spanned the entire alignment, we then filtered out any alignments that aligned more than once to the reference and filtered alignments that were shorter than 3,500 nucleotides (98 per cent of the length of the reference genome), overall keeping 38 per cent of 61,248 reads. We further removed alignments that resulted in a minus strand alignment, i.e. were the reverse complement of the genome.

As described later, our model relies on single-nucleotide variation (SNV) counts and in particular categorizes them as synonymous or non-synonymous. We thus excluded both the untranslated regions of the genomes (which are very short and account for less than 10 per cent of the genome) and indels from our analysis, as their biological effects are more complex to analyze. Notably, we ignore large genomic modifications and focus only on SNVs to estimate the point mutation rate.

### Evolutionary model

**Figure 1.**
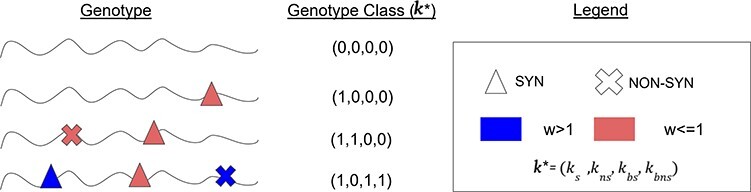
The illustration of genotype classes. Our evolutionary model assigns genotypes to genotype classes. Each genotype class *k* is a 6-tuple that represents all genomes with the same number of non-beneficial synonymous SNVs that derive from the initial population, non-beneficial non-synonymous SNVs from the initial population, non-beneficial synonymous SNVs, non-beneficial non-synonymous SNVs, beneficial synonymous SNVs, and beneficial non-synonymous SNVs. For simplicity, simplified genotype classes (k*) consisting of only 4-tuples representing the last four types of mutations (i.e. excluding the initial founder population SNVs) are represented in the figure. SNVs of the same type are interchangeable and are assumed to have the same fitness effect (*w*). Genotype classes allow our model to be computationally tractable while still giving a detailed account of the evolutionary dynamics.

Instead of separately considering each SNV in the genome, we label each SNV as belonging to one of the following types based on whether a mutation at a site is (1) synonymous or not, (2) beneficial or non-beneficial, and (3) a part of the founding population or not. We group genotypes by the number of each of these mutation types into genotype classes (illustrated in Fig. 1). Monitoring just the genotype classes, rather than the genotypes themselves, reduces the number of model parameters to only ten and makes our model computationally tractable.

#### Initial founding population

Our experimental conditions were designed to ensure a homogeneous initial population, derived from a plaque. However, the generation of this initial population still included multiple replication cycles at slightly different conditions than our experiment, such as higher MOI. We thus directly model the limited diversity present in the initial population. We assume that the average number of synonymous SNVs per genotype in the initial population is ${M_s}$ and the average number of non-synonymous SNVs per genotype in the initial population is ${M_{ns}}$. We assume that the number of SNVs in the initial population is $M \sim Poi\left( {{M_s} + {M_{ns}}} \right)$ and for simplicity also assume that this plaque-derived founding population does not contain beneficial SNVs. Therefore, the number of synonymous ${k_{Is}}$ and non-synonymous ${k_{Ins}}{\ }$mutations per individual in the initial population is as follows:


(1A) $${k_{Is}} \sim {\rm{Binomial}}\left( {M,\;\frac{{{M_s}}}{{{M_s} + {M_{ns}}}}} \right),$$

(1B) $${k_{Ins}} = M - {k_{Is}},$$

#### Evolution during serial passaging

We model the evolutionary dynamics using a Wright–Fisher framework with a constant population size $N$ and non-overlapping generations. We follow the frequency of genotypes with exactly ${k_{Is}}$ non-beneficial synonymous SNVs that derive from the initial population, ${k_{Ins}}$ non-beneficial non-synonymous SNVs from the initial population, ${k_s}$ non-beneficial synonymous SNVs, ${k_{ns}}$ non-beneficial non-synonymous SNVs, ${k_{bs}}$ beneficial synonymous SNVs, and ${k_{bns}}$ beneficial non-synonymous SNVs, such that each genotype can be classified into a class *k = (*${k_{Is}},{k_{Ins}},{k_s},{k_{ns}}$, ${k_{bs}}$, ${k_{bns}}),{\ }{k_i} \ge 0$.

##### Mutation.

We assume that the number of new SNVs per genotype per replication cycle is Poisson distributed with the expected value *U*, which is the mutation rate: the expected number of mutations per genome per replication cycle (Tenaillon et al. 1999). The probability that an SNV is synonymous or non-synonymous is given by ${p_s}$ and ${p_{ns}} = 1 - {p_s}$, respectively. The probability that a synonymous or non-synonymous SNV is beneficial is ${p_{bs}}$ and ${p_{bns}}$, respectively. Thus, the number of new SNVs per genotype is given by


(2) $$u \sim Poi\left( U \right),\,\,\Delta = \left( {\Delta s,{\ }\Delta ns,{\ }\Delta bs,{\ }\Delta bns} \right) \sim Multinomial\left( {u,{\ }{p_\Delta }} \right),$$

where *u* is the total number of new SNVs in one individual, $\Delta i$ is the number of new SNVs of type *i*, and ${p_\Delta } = \left( {{p_s} - {p_{bs}},{\ }{p_{ns}} - {p_{bns}},{\ }{p_{bs}},{\ }{p_{bns}}} \right)$ (${p_\Delta }$ sums to 1). Thus, the change in *f_k_*, the frequency of genotype class *k*, due to mutation is given by


(3) $$f_{k}^{mut} = \sum\nolimits_{g} f_{g}\ \cdot P(\Delta _{k - g}),$$

where *g* is an index over all existing genotype classes that can mutate to genotype class *k*; ${\Delta _{k - g}}$ is the difference in the number of mutations of the various types between genotype class *k* and *g*; and *P(*${\Delta _{k - g}}$) is the Poisson-multinomial probability mass function ($eq.{\ }2$). Since the Poisson-multinomial distribution has infinite support, we truncate the distribution by setting $P({\Delta _{k - g}}) = 0$ if $P({\Delta _{k - g}}) \lt 1/\left( {100N} \right)$. We neglect the effect of recombination due to low MOI.

##### Fitness.

For simplicity, we assume a single value for the fitness effect of synonymous non-beneficial SNVs, ${w_s}$, and a single value for fitness effect of non-synonymous non-beneficial SNVs, ${w_{ns}}$. Beneficial SNVs (synonymous or non-synonymous) are modeled separately, and the fitness effect of beneficial SNVs (synonymous or non-synonymous) is ${w_b}$.

##### Initial founder population fitness.

Since the initial population evolved under possibly different selection pressures (described previously), we allow SNVs in the initial population to have different fitness effects, but we assume that the log-fitness of these initial SNVs is correlated with the log-fitness of non-beneficial SNVs in the experiment, with correlation coefficient $\delta $: here, $\delta $ = 0 implies that the initial population SNVs are neutral under the experimental conditions; $\delta $ = 1 implies that the initial population SNVs fitness effects are the same as that of the non-beneficial SNVs; and $\delta \gt 1$ implies that the SNVs in the initial population are more deleterious than later non-beneficial SNVs.

##### Epistasis.

We also model the potential interaction of multiple beneficial SNVs by introducing an epistasis parameter *η*: when *η* > 1, there is positive epistasis; when 0.5 < *η* < 1, there is negative epistasis; when *η* < 0.5, there is sign epistasis; and when *η* < 0, there is reciprocal sign epistasis so that the effect of two beneficial mutations is deleterious.

##### Fitness of genotype classes.

We assume multiplicative fitness. Therefore, the fitness of genotype class *k =*$({k_{Is}},{k_{Ins}},{k_s},{k_{ns}}$, ${k_{bs}}$, ${k_{bns}})$ is


(4) $${w_{k{\ }}} = {w_s}^{\delta \cdot {k_{Is}}} \cdot {w_{ns}}^{\delta \cdot {k_{Ins}}} \cdot {w_s}^{{k_s}} \cdot {w_{ns}}^{{k_{ns}}} \cdot {w_b}^{\eta \cdot \left( {{k_{bns}} + {k_{bs}}} \right)},$$

where *η =1* if ${k_{bns}} + {k_{bs}} \lt 2$.

Thus, the effect of natural selection on genotype frequencies is given by


(5) $${f_k}^{sel} = {w_k} \cdot {f_k}^{mut}/\overline w ,$$

where $\overline w = \mathop \sum \limits_j {w_j} \cdot {f_j}^{mut}$ is the population mean fitness.

##### Random sampling.

Serial passaging that includes sampling progeny virus for the next passage is modeled by assuming random sampling. Thus, the number of virions of each genotype class after sampling is given by $n = \left\{ {{n_k}} \right\} \sim Multinomial\left( {N,{f^{sel}}} \right)$, where ${f^{sel}} = \left\{ {{f_k}^{sel}} \right\}{\ }$and *k* is an index over all existing genotype classes. Accordingly, the frequency of genotype class *k* in the next generation is


(6) $${f_k}^{\prime}= {n_k}/N.$$

We simulate the stochastic genotype frequency dynamics in the experiment by iterating equations 1–6 for multiple generations.

##### Sequencing process.

Because only a sample of genomes is sequenced, and sequencing is error prone, we model sequencing errors and sampling. The frequency $\widehat {{f_k}}$ of genotype class *k* after sampling and sequencing is


$$\widetilde{f}_{k} = \sum_{g} f_{g}\ \cdot P( \Delta _{k - g}),\\[-32pt]$$

$$\tilde u \sim Poi\left( {{N_s}er{r_{seq}}} \right),\\[-32pt]$$

$$\Delta = \left({\Delta s, \Delta ns, \Delta bs, \Delta bns} \right) \sim Multinomial\left(\widetilde{u}, {p_\Delta } \right),\\[-32pt]$$

$$\left\{ {\widetilde {{n_k}}} \right\} \sim Multinomial\left( {{N_s},\widetilde {{f_k}}} \right),\\[-32pt]$$

$$\widehat {{f_k}} = \widetilde {{n_k}}/{N_s},$$

where ${N_s}$ is the sample sequencing coverage (see later) and $er{r_{seq}}$ is the expected average sequencing error set at $er{r_{seq}}$ = 5 · 10^−5^ errors per base, based on the reported LoopSeq error rate (Callahan et al. 2021). We further tested $er{r_{seq}}$ = 5 · 10^−4^ errors per base to assess the robustness of our method to a higher error rate.

#### Experimental parameter values

A viral population size $N = 2\cdot{10^7}$ was assumed corresponding to the experimental setup (see earlier section). Each line and passage had different sequencing coverage *N_s_* (mean = 2,589, standard deviation = 1,195, minimum = 1,309, and maximum = 4,976; see Table S1 for details). The probability that an SNV is synonymous is ${p_s} = 0.28$, corresponding to the possible point synonymous and non-synonymous SNVs in the MS2 reference genome. Other parameter values were inferred from experimental data (see next section).

### Likelihood-free Bayesian inference

#### Summary statistics

We applied three summary statistics: short-reads summary statistic (SR), long-reads summary statistic (LR), and labeled long-reads summary statistic (L-LR). The simplest, SR, is a vector of the average number of synonymous and non-synonymous SNVs per genotype at each passage *t*. Thus, SR is a vector with two entries per passage and six entries in total.

**Figure 2.**
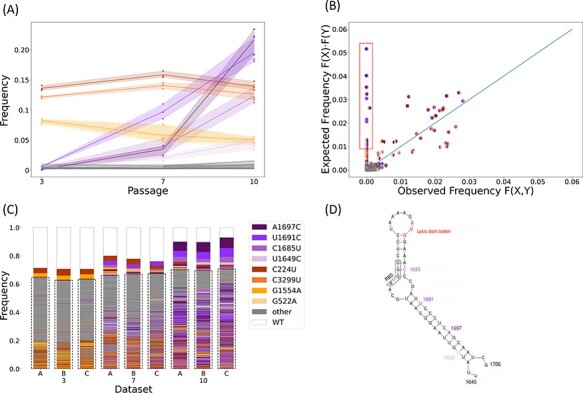
SNV and genome frequencies across time. (A) Markers show SNV frequencies in the three replicas, with high-frequency SNVs in purple, founder SNVs in brown-orange, and all other low-frequency SNVs in gray. The shaded area illustrates the range of observed values. (B) Observed versus expected frequencies of pairs of SNVs. For a pair of SNVs denoted by X and Y, *F(X)* and *F(Y)* are their frequencies across all genotypes, respectively; *F(X)F(Y)* is the expected frequency of the pair assuming independence; and *F(X, Y)* is the observed frequency of the pair across all genotype; frequencies are taken from all three replicas at passage 10. The diagonal line represents the independence of the pair of SNVs, i.e. *F(X, Y) = F(X)F(Y)*. Founder SNV pairs were excluded from this analysis. Pairs of beneficial SNVs are surrounded by a red box. (C) Genetic diversity for each replica and passage. When a genome contains more than one high-frequency SNV, the color of its box is defined by the first SNV in the order of appearance in the legend. The genomes are ordered by their frequency in each dataset and the number of SNVs within them. Rare genotypes are defined as present in less than 0.5 per cent of a sample and are boxed with a dashed line. (D) The RNA structure where all beneficial SNVs occur is inferred using *Mfold* (Zuker 2003) and in line with the experimentally resolved structure (Dai et al. 2017). This region spans the end of the coat gene and the overlapping lysis gene. All beneficial SNVs are synonymous with respect to the coat gene and non-synonymous with respect to the lysis gene. ‘RBS’ marks the ribosomal-binding site for the lysis gene.

Using LoopSeq long-read sequencing, which covers the entire MS2 genome, we could use a more informative summary statistic, LR, which counts the frequency of genotypes containing exactly ${k_{Is}} + {k_s} + {k_{bs}}$ synonymous SNVs and ${k_{Ins}} + {k_{ns}} + {k_{bns}}$ non-synonymous SNVs, respectively, at passage *t*. We counted up to ten SNVs of each type, producing 66 possible SNV combinations per passage. We also included SR in LR, producing a vector with 68 entries per passage and 204 entries in total.

We used a similar but more informative summary statistic, L-LR, which includes the number of beneficial SNVs. We labeled four SNVs as beneficial: they were all rare at passage 3 but reached a frequency higher than 3 per cent in all three lines by passage 10 (Fig. 2A, purple lines). Assuming that the per-base mutation rate is at most 0.001 (Drake 1993), a neutral SNV is expected to be at a frequency of about 1 per cent by passage 10. Labeling the beneficial SNVs allowed us to obtain the frequencies of genotypes containing exactly ${k_{Is}} + {k_s}$ synonymous non-beneficial SNVs, ${k_{bs}}$ synonymous beneficial SNVs, ${k_{Ins}} + {k_{ns}}$ non-synonymous non-beneficial SNVs, and ${k_{bns}}$ non-synonymous beneficial SNVs at each passage *t*. We counted up to ten SNVs of each of the four types, producing 1,001 frequency values per passage. Including SR in L-LR produced a vector with 3,009 entries in total.

#### Prior distributions

We assumed uniform (noninformative) prior distributions with ranges set based on current estimates in the literature (Table 1). The prior distribution of ${w_b}$ is much wider than the estimates in the literature to account for the rapid increase in frequencies of some SNVs we observe in the empirical data (Fig. 4B). Prior distributions for ${w_s}$ and ${w_{ns}}$ are wide to avoid bias in the posterior. Prior distributions of ${M_s}$ and ${M_{ns}}$ are based on experimental sequencing data from three different MS2 plaque sequences (data not shown).

**Table 1.** Model parameters.

Description	Symbol	Prior distribution
Average non-beneficial synonymous fitness effect	${w_s}$	Uniform (0.1, 1)
Average non-beneficial non-synonymous fitness effect	${w_{ns}}$	Uniform (0.1, 1)
Average beneficial fitness effect	${w_b}$	Uniform (1, 3) (Sanjuán, Moya, and Elena 2004)
Beneficial synonymous SNV probability	${p_{bs}}$	Uniform (0, 0.01) (Sanjuán, Moya, and Elena 2004)
Beneficial non-synonymous SNV probability	${p_{bns}}$	Uniform (0, 0.01) (Sanjuán, Moya, and Elena 2004)
Average number of synonymous SNVs per genotype in the initial population	${M_s}$	Uniform (0.4, 0.6)
Average number of non-synonymous SNVs per genotype in the initial population	${M_{ns}}$	Uniform (0.7, 0.9)
Initial log-fitness correlation	$\delta $	Uniform (0, 2)
Epistasis	$\eta $	Uniform (−1, 3)
Genome-wide mutation rate	*U*	Log-Uniform (−4, 0.3) (Sanjuán 2010)

#### Sequential neural posterior estimation

We used a recently developed neural-network-assisted likelihood-free inference method, *sequential neural posterior estimation* (SNPE) (Greenberg, et al. 2019) or, specifically, the SNPE-C implementation in the Python package *sbi* (Tejero-Cantero et al. 2020). SNPE has been recently applied for inferring the formation rate and fitness effect of copy number variation in populations of yeast evolving under nutrient limitation in a chemostat (Avecilla et al. 2022). Briefly, SNPE trains an artificial neural network on a training set of parameters (generated from the prior distribution) and simulated data (generated from the evolutionary model given parameter values) to estimate the joint density of model parameters and data (conditioned on the prior distribution). This joint density is effectively an *amortized posterior distribution* of the model parameters, which can be evaluated for specific observed data. Amortization allows the evaluation of the posterior distribution for each experimental observation (i.e. replicate or line) without needing to re-train the neural network (in contrast to Markov chain Monte Carlo based approaches that require a new run of the algorithm to infer a posterior from a new observation). As the neural density estimator, we used a *masked autoregressive flow* (Papamakarios, Pavlakou, and Murray 2017), which uses a stack of autoregressive models (specifically, *masked autoencoders for distribution estimation*; Germain, Gregor, and Murray 2015) to model a sequence of transformations on random variables.

##### Ensemble SNPE.

We further extend SNPE to the *ensemble SNPE* by averaging the posterior distribution estimated by *eight* density estimators, each independently trained with non-overlapping training sets of 10,000 simulations sampled from the same prior. We compare the performance of the ensemble SNPE to *individual SNPE*, which we trained on a training set of 80,000 simulations.

#### Approximate Bayesian computation with rejection sampling

We compared SNPE with a classical likelihood-free inference method, *approximate Bayesian computation with rejection sampling* (REJ-ABC) (Pritchard et al. 1999; Sunnåker et al. 2013). ABC is used to approximate posterior distributions when an underlying likelihood function is analytically and/or computationally intractable. In REJ-ABC, parameter sets are independently sampled from the prior; a simulation is run for each parameter set; the distance between simulation results and the observed data is computed; parameter sets that produced the lowest *ε*-percentile distances are accepted (while the rest are rejected); and the posterior distribution is estimated from the accepted parameter sets. We used 80,000 parameter sets and simulations (the same simulations that we used to train SNPE) to produce the REJ-ABC posterior estimation. As a distance function, we used the root mean square error between the simulated and observed summary statistics. We produced posterior distributions using acceptance rates of *ε *= 0.1 per cent, 1 per cent, and 5 per cent.

#### Parameter estimates and posterior predictive checks

To obtain a marginal maximum a-posteriori (MAP) estimate, we sample from the posterior distribution and construct a histogram with 100 bins. We then estimate the parameter as the center of the most frequent bin. We calculated the highest density intervals (HDIs) using the Python package *ArviZ* (Kumar et al. 2019). We performed posterior predictive checks by simulating synthetic data with parameters sampled from the posterior and comparing it to the observed data.

#### Flexible inference from time series

We also compared SNPE to a previous method developed by some of us called *flexible inference from time series* (*FITS*) (Zinger et al. 2019). In contrast to the above-mentioned REJ-ABC and SNPE, FITS applies a single-locus Wright–Fisher model to all synonymous SNVs, assuming that they are all neutral, and uses rejection sampling to approximate the posterior distribution of model parameters. Thus, FITS does not use the evolutionary model, prior distributions, and summary statistics described earlier.

#### Premature stop codons

We counted the number of premature stop codons across the genome in all reads from all passages and replicas and divided by the number of possible SNVs that could have resulted in a premature stop codon. Assuming premature stop codons are lethal, under a mutation–selection balance, this ratio is expected to be equal to the mutation rate. The problem with this approach is that the observed premature stop codons due to mutations cannot be distinguished from sequencing errors (with a rate estimated at $er{r_{seq}}$ =5 · 10^−5^, see earlier) and so this method only provides an upper bound on the estimated mutation rate.

## Results

### Individual SNV frequencies suggest positive selection

We began by inspecting the haplotype sequencing data, which was derived from passages 3, 7, and 10 from three independent biological replicas (A, B, and C). We first examined the SNV frequencies, focusing on those segregating at frequencies more than 3 per cent in all three replicas by the end of the experiment. Four SNVs were at a frequency more than 5 per cent already at passage 3 and remained at more or less constant frequencies throughout the experiment. Hence, we assumed that they reflect standing variation in the initial founding population (Methods) and denote them as *founder SNVs* (Fig. 2A). Conversely, we noted a set of four SNVs that increased dramatically in frequency, rising from less than 1 per cent to frequencies ranging between ∼5 per cent and ∼20 per cent in all three replicas (Fig. 2A). This increase suggests that these four SNVs are under positive selection, and we thus denote them as *beneficial SNVs*.

We next examined haplotype composition, starting with pairs of SNVs. We compared the observed frequency of each SNV pair with its expected frequency assuming that they are pairwise independent, which is the product of their individual frequencies (Fig. 2B). We find that the four founder SNVs appear almost exclusively in two specific pairs (C3299T with C224T and G1554A with G522A, Fig. 2A, not shown in 2B). In contrast, the four beneficial SNVs rarely or never appear on the same genome (only one pair appears in one genome), exhibiting negative linkage disequilibrium. The low observed frequency of the beneficial SNV pairs can have two explanations: negative or sign epistasis between the beneficial SNVs and a combination of strong selection and low mutation rate, which together do not allow enough time for the accumulation of two beneficial SNVs on the same genome.

Next, we examined the genome diversity across replicas and passages. We noted that by passage 10, 55 per cent, 54 per cent, and 65 per cent of the genomes had a beneficial SNV in replicas A, B, and C, respectively. We also observed high genome diversity across all replicas and all passages (Fig. 2C). Indeed, except for the wildtype genome, which was present in 10 per cent, 10 per cent, and 7 per cent in replicas A, B, and C, respectively, no genotype exceeded 7 per cent, and 70 per cent of genotypes were rare, i.e. present at frequencies lower than 0.5 per cent. For example, the beneficial SNV A1697C reached a frequency of ∼20 per cent by passage 10, but no single genotype bearing A1697C had a frequency more than 7 per cent (Fig. S6). This high level of diversity, including the abundance of rare genotypes, is suggestive of a high mutation rate, the occurrence of soft selective sweeps, and possible epistatic effects. We therefore developed a framework to jointly estimate the various evolutionary parameters from our time-series haplotype data (Methods).

### Validation of inference method on synthetic data

To validate the performance of our method, we simulated 2,000 synthetic datasets using the evolutionary model and a set of known parameter values (denoted as the ‘true’ parameter values). We tested three summary statistics, each with more information than the previous one (SR, LR, and L-LR; see Methods).

**Figure 3.**
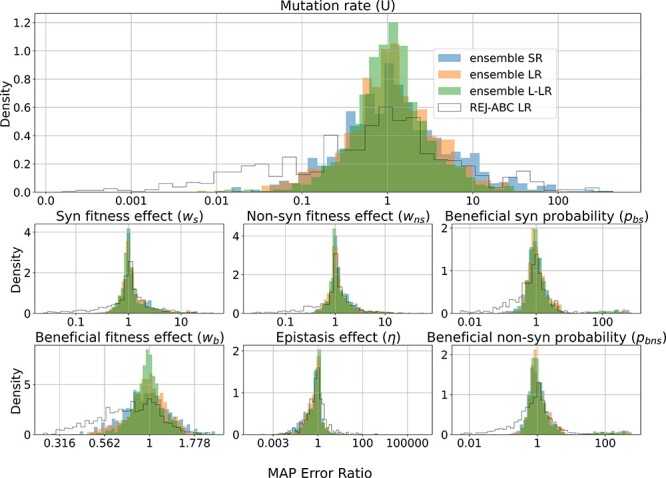
Parameter estimation accuracy on synthetic data. Ratio of MAP estimate and true parameter value on 2,000 synthetic datasets. Inferences used SNPE with three different summary statistics (SR, blue; LR, orange; and L-LR, green) and REJ-ABC (LR, white). See Fig. S10 for a direct comparison of true vs. estimated parameter values and Fig. S13 for parameter values that lead to estimation errors.

#### Coverage

We first measured the *coverage*, defined as the proportion of inferred posterior distributions in which the credible interval for a parameter contains the true parameter value (Prangle et al. 2014). We found that *individual SNPE* (a single neural density estimator trained on 80,000 simulations) produced posteriors that were overconfident, i.e. the 95 per cent HDI contained the true parameter in less than 95 per cent of the test cases (Fig. S1).

Indeed, it has been suggested that simulation-based inference methods may produce overconfident posterior approximations and that this issue may be mitigated by using ensembles of estimators (Hermans et al. 2022). We therefore extended the individual SNPE to an *ensemble SNPE* that comprises *eight* individual SNPEs, each independently trained with non-overlapping training set (with a total simulation budget fixed at 80,000 simulations). We found that while individual SNPE mostly produces overconfident posteriors, the ensemble SNPE produces posteriors that are less confident, i.e. has superior coverage compared to the individual SNPE (Fig. S1).

#### Parameter estimate accuracy

We compared our MAP estimates, which are point estimates of model parameters, with those of a simpler method, REJ-ABC. The latter was run with a 1 per cent acceptance rate using the same dataset of 80,000 simulations used to train the ensemble SNPE (Methods). REJ-ABC produced the largest MAP errors, on average, and did not seem to improve with more informative summary statistics (Fig. S2). For the SNPE inference methods, we found that more informative summary statistics produced similar or smaller MAP errors (Fig. S2). Ensemble SNPE performed similarly to the individual SNPE (Fig. S2). We chose to henceforth focus on ensemble SNPE because it had better coverage in most cases, especially for the mutation rate (Fig. S1).

As expected, the more informative the summary statistic, the narrower the posterior distributions, and thus, the higher the information gain calculated by the Kullback–Leibler divergence (Fig. S3). The MAP error ratios of the ensemble SNPE were centered around zero, suggesting that they were unbiased (Figs 3 and S10). We also observed a slight improvement in the MAP error ratio when increasing the information in the summary statistics. All ensemble SNPE produced lower absolute MAP error ratios compared with REJ-ABC (Fig. 3).

**Table 2.** Mutation rate estimates. MAP estimates and 95 per cent HDIs of the posterior distributions of the genomic mutation rate *U* from Fig. 4A. Note that all estimates are similar, but ensemble SNPE produced HDI that is two orders of magnitude narrower than FITS (single-locus Wright–Fisher model with rejection sampling applied to all synonymous SNVs).

Method	MAP estimate of *U*	95% HDI
FITS	0.197	0.0004–0.796
Premature stop codon frequency^a^	≤0.187	N/A
Ensemble SNPE with SR	0.161	0.013–1.396
Ensemble SNPE with LR	0.221	0.014–1.548
Ensemble SNPE with L-LR	0.194	0.056–0.73

a Frequency of premature stop codons does not produce an HDI; rather, it produces an upper bound on the mutation rate and therefore reflects either the mutation rate or the sequencing error.

### Inference of evolutionary parameters from empirical data

#### Mutation rate

We next applied the ensemble SNPE to the haplotype data from the MS2 experiment (Fig. 2A), averaging the estimated posteriors over the three experimental replicas, and assuming sequencing errors at a rate of 5 · 10^−5^ per base, as reported by Loop Genomics (Callahan et al. 2021). We found that the ensemble SNPE with the SR and LR summary statistics produced similar posteriors, whereas using the L-LR summary statistic produced a narrower posterior distribution and was able to reproduce the dynamics of the evolving population (Fig. S8). All MAP estimates of the mutation rate *U* are between 0.15 and 0.2 mutations per genome per replication cycle (Table 2) and the widest HDI 95 per cent is between 0.016 and 1.6. Given a genome length of 3,569 bases, this corresponds to between 4.2 · 10^−5^ and 5.6 · 10^−5^ SNVs per base per replication cycle.

#### Comparison to other methods

**Figure 4.**
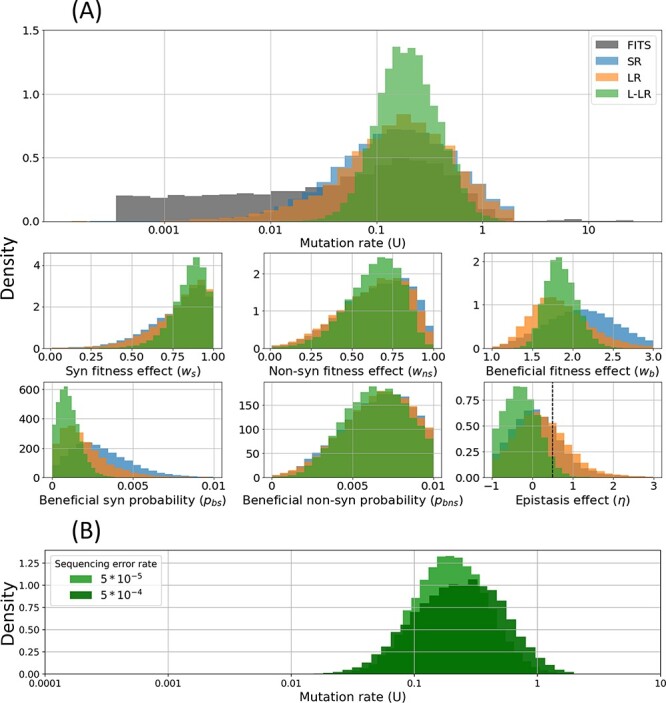
Posterior distributions of model parameters inferred for MS2. (A) Posterior distributions of the mutation rate using three summary statistics for the mutation rate produce a substantially narrower distribution compared to FITS (single-locus Wright–Fisher model with rejection sampling applied to all synonymous SNVs). Shown are marginal posteriors of model parameters using ensemble SNPE with three different summary statistics. In most cases, the L-LR produces a narrower posterior, and generally all posteriors agree. For epistasis, the left of the dotted line are values indicating sign epistasis. (B) The marginal posterior distribution of the mutation rate *U* inferred with ensemble SNPE with the L-LR summary statistic compared to an estimate that assumes a tenfold increase in the sequencing error rate. See Fig. S11 for joint posterior distributions of the mutation rate with other model parameters.

We next compared our ensemble SNPE inference method to two alternative mutation rate inference methods: one based on the frequency of premature stop codons and the other FITS (Zinger et al. 2019), which is based on changes in the frequency of neutral SNVs (Methods). We found that the MAP estimates from SNPE are in line with the frequency of premature stop codons, which is highly susceptible to sequencing errors and is therefore only an upper bound. Furthermore, SNPE produces a substantially narrower posterior distribution compared to FITS, regardless of the summary statistic (Fig. 4A and Table 2).

#### Sequencing error rate

Reassuringly, when assuming a tenfold increase in the sequence error rate (5 · 10^−4^), inference with the L-LR summary statistic was robust. Importantly, the MAP estimate of the mutation rate only slightly shifted from 0.194 (95 per cent HDI, 0.056–0.73) to 0.282 (0.037–1.26). Overall, the posterior distribution of the mutation rate and most other parameters remained similar (Figs 4C and S9) although the mode of the posterior distributions of non-synonymous fitness effect (*w_ns_*) and beneficial non-synonymous probability (*p_bns_*) shifted under a higher error rate. However, using the SR and LR summary statistics produced wider posterior distributions for most model parameters (Figs S4–S5). These results underscore the inherent difficulty in inferring mutation rates and fitness effects with error-prone short-read sequencing approaches and suggest that there is a much-added value in labeling beneficial SNVs when possible.

#### Fitness effects

We report estimates from the ensemble SNPE with L-LR summary statistics given its superior performance (Table S2). The estimated non-beneficial synonymous mutation fitness effect to be about 0.9 (95 per cent HDI, 0.651–1), implying that most synonymous mutations are neutral or slightly deleterious (Sanjuán, Moya, and Elena 2004; Cuevas, Domingo-Calap, and Sanjuán 2012). The estimated non-beneficial non-synonymous mutation fitness effect is about 0.7 (95 per cent HDI, 0.311–0.922; Fig. 4B). This estimate implicitly averages slightly deleterious, deleterious, and lethal mutations. The fitness effect of beneficial SNVs is estimated at about 1.8 (95 per cent HDI, 1.5–2.2).

#### Epistasis

We also estimate the epistasis between beneficial SNVs. The epistasis parameter *η* is estimated at about −0.3 (95 per cent HDI, −1 to 0.443), which implies sign epistasis and likely even reciprocal sign epistasis, in which the combined effect of two beneficial mutations is deleterious. To further validate this estimate, we used simulations to determine if the observation that pairs of beneficial SNVs rarely reside on the same genome (Fig. 2B) could be explained by a two-locus bi-allelic Wright–Fisher model with just mutation, genetic drift, and strong selection, but without epistasis. Our simulation results strongly suggest that such a model cannot explain the experimental results (Fig. S7), providing further support for sign epistasis between beneficial SNVs in MS2.

## Discussion

Both selection and sequencing errors can complicate the estimation of mutation rates from sequencing data (Peck, Lauring, and Sullivan 2018; Gelbart et al. 2020). Nevertheless, we show that our approach, which applies Bayesian inference to evolutionary experimental data using simulations and artificial neural networks, performs well on synthetic simulated data and allows us for the first time to estimate the point mutation rate of MS2 with a narrow confidence interval. In contrast to methods based on single-locus models, we set out to develop a multi-locus model that can account for background selection on full viral genomes as well as for standing variation in the founding population. This represents a challenge: even with the short genome of the MS2 virus, a high mutation rate leads to high genetic diversity (Fig. 3D), which makes the number of possible haplotypes huge (∼4^3500^). Thus, our model groups genomes by the number of mutant alleles they accumulated in several mutation types (Fig. 1). This model is simple enough to simulate efficiently but complex enough to capture the genome frequency dynamics. Furthermore, SNPE is also computationally more efficient than sampling methods such as REJ-ABC due to its application of neural density estimators to directly approximate the posterior distribution using state-of-the-art deep-learning algorithms and hardware acceleration; furthermore, amortization allows us to estimate a posterior distribution for new datasets without running the inference algorithm again, which cannot be done with sampling methods (Avecilla et al. 2022).

Our estimate of the MS2 mutation rate is roughly *U = *0.2 mutations per genome per replication cycle (95 per cent HDI, 0.056–0.73; Table 2). Reassuringly, we find that our method is robust to sequencing errors, which present a challenge for any method that relies on rare SNV frequencies (Acevedo, Brodsky, and Andino 2014; Gelbart et al. 2020). Compared with a close relative of MS2, Qβ, our inferred mutation rate is an order-of-magnitude lower than the early estimates of the mutation rate of Qβ, which were around *U = 6.5* (Batschelet, Domingo, and Weissmann 1976; Domingo, Flavell, and Weissmann 1976). These studies quantified the mutation rate of one specific nucleotide at position 40 of the genome of Qβ. Therefore, their estimates may be biased for that specific site, while our estimates represent a genome-wide mutation rate. A more recent estimate of the Qβ mutation rate at about *U = *0.08 was based on phenotypic scores (Garcia-Villada, Drake, and Hughes 2012). Another study (Bradwell et al. 2013) corrected this estimate using the distribution of fitness effects obtained previously for this phage (Domingo-Calap, Cuevas, and Sanjuán 2009) and estimated a mutation rate of *U = 0.6*. This estimate is three times higher than our estimate and within our estimated HDI. Importantly, the difference between mutation rate estimates for Qβ and MS2 may represent true biological differences between the two phages; indeed, their genomes share only ∼45 per cent identity.

The inferred mutation rate, together with the very large population size ($\theta $*=N· U/3,500 = 1,142*), puts this virus population in the regime of rapid adaptation unlimited by mutation (Feder, Pennings, and Petrov 2021). In this regime, soft sweeps are expected to occur and leave their mark on the genome. Indeed, we find evidence for multiple beneficial SNVs segregating in the population in multiple genotypes (Fig. 2). It would be interesting to estimate $\theta $ from SNV polymorphism dynamics (Pennings and Hermisson 2006; Feder, Pennings, and Petrov 2021) to see if it agrees with our inference results, which directly estimates the mutation rate, as the population size is known in our experiment.

The posterior distribution of the non-beneficial non-synonymous fitness effect *w_ns_* is wide (Table S2). On the one hand, it is reassuring that it is clearly different from the posterior of the non-beneficial synonymous fitness effect *w_s_* (Fig. 3). However, the MAP estimate of 0.7 for *w_ns_* is higher than previous estimates in the literature that found a high frequency of lethal SNVs (Sanjuán, Moya, and Elena 2004; Cuevas, Domingo-Calap, and Sanjuán 2012). This difference may be explained by our use of a single fitness effect parameter to describe all non-beneficial non-synonymous SNVs, the distribution of which is likely bi-modal, with one ‘peak’ for lethal mutations and one ‘peak’ for slightly deleterious mutations (Sanjuán, Moya, and Elena 2004; Domingo-Calap, Cuevas, and Sanjuán 2009; Cuevas, Domingo-Calap, and Sanjuán 2012).

Epistatic effects are notoriously difficult to estimate from empirical data as they require departing from simple linear models, and more complex models require more data and stronger effect sizes to achieve statistical power (Tromas and Elena 2010). We first suspected epistatic effects when we observed that the beneficial SNVs are rarely found together on the same genome (Fig. 2B). To address this, we added an epistasis parameter to our model (Equation 4). All our inferences estimated the epistasis parameter to be less than 0.5, implying sign epistasis (Fig. 4). Notably, this inference relied on the L-LR summary statistic, which uses labeled beneficial SNVs.

Interestingly, all four beneficial SNVs reside on the same RNA structure (Fig. 2D) and are close to the ribosomal binding site of the lysis protein. These SNVs are synonymous for the coat protein, and three are non-synonymous for the lysis protein, whereas the fourth is not within the lysis gene. This may suggest that these beneficial SNVs either affect lysis translation or change the lysis protein itself. Moreover, a previous work has inferred that each of the single SNVs increases lysis expression, whereas a double mutant creates an unstable RNA structure that does not allow for lysis protein production and is therefore deleterious (Betancourt 2009). Increased lysis expression in single mutants may provide a strong fitness benefit due to the completion of two replication cycles in a single passage (this effect may slightly shift our estimates, see Fig. S14). An alternative explanation for the beneficial effect of these SNVs is that these mutations decrease lysis expression, which gives the genome more time to replicate and create more progeny.

We next discuss the applicability of our method to other organisms and experimental setups. Our method includes several parts: an evolutionary model, summary statistics, and an inference framework that uses simulations and neural networks to approximate a posterior distribution over the model parameters. Our best-performing summary statistics (LR and L-LR) require long-read sequencing data. This restricts its application mostly to study either laboratory evolution or within-host virus evolution. The inference framework (SNPE) has recently been applied to yeast growing in nutrient-limited chemostats, with a simpler model focusing on the frequency of cells with increased copy number variation (Avecilla et al. 2022). Similar approaches can be applied to other microbial species (e.g. *E. coli*) in evolutionary experiments and can include additional factors such as fluctuating population sizes and structured populations.

### Conclusions

Here, we applied recent innovations in likelihood-free inference that use neural-network density estimators to directly approximate the posterior distribution rather than sampling from it and long-read sequencing that covers the full length of a viral genome. This allowed us to efficiently and precisely infer the joint posterior distributions of the parameters governing the genome frequency dynamics in an evolutionary experiment with the MS2 bacteriophage, thereby estimating its mutation rate and fitness effects.

## Supplementary Material

vead033_Supp

## Data Availability

The source code is available at https://github.com/Stern-Lab/ms2-mutation-rate. Sequencing data are available at https://www.ncbi.nlm.nih.gov/sra/PRJNA902661. Additional data are available at https://zenodo.org/record/7486851.
