# Supplementary material for: Mutation rate, selection, and epistasis inferred from RNA virus haplotypes via neural posterior estimation
Source: Virus Evol. 2023 May 20;9(1):vead033. doi: 10.1093/ve/vead033 (PMC10256221; doi:10.1093/ve/vead033)
Supplement: vead033_Supp [file vead033_supp.zip › suppl_data/Supplementary materials.docx]

# Supplementary materials

Mutation rate, selection, and epistasis inferred from RNA virus haplotypes via neural posterior estimation

Itamar Caspi^1^, Moran Meir^1^, Nadav Ben Nun^2,3^, Reem Abu Rass^1^, Uri Yakhini^1,2^, Adi Stern^1,2,^*, Yoav Ram^2,3,^*

^1^ Shmunis School of Biomedicine and Cancer Research, Faculty of Life Sciences, Tel Aviv University

^2^ Edmond J. Safra Center for Bioinformatics, Tel Aviv University

^3^ School of Zoology, Faculty of Life Sciences, Tel Aviv University

* Corresponding authors: AS: [sternadi@tauex.tau.ac.il](mailto:sternadi1@gmail.com), YR: [yoavram@tauex.tau.ac.il](mailto:yoavram@tauex.tau.ac.il)

# May 8, 2023

**Supplementary Table 1. Sequencing Coverage.** Number of synthetic long reads in each dataset after filtering (see Methods).

| **Replica** | **Passage** | **Sequencing Coverage** |
| --- | --- | --- |
| A | 3 | 1343 |
|  | 7 | 4414 |
|  | 10 | 1552 |
| B | 3 | 2347 |
|  | 7 | 2604 |
|  | 10 | 1230 |
| C | 3 | 2446 |
|  | 7 | 1333 |
|  | 10 | 3350 |

**Supplementary Table 2. Parameters estimates for ensemble SNPE.**

| Parameter | Notation | Short-reads summary statistic | | Long-reads summary statistic | | Labeled long-reads summary statistic | |
| --- | --- | --- | --- | --- | --- | --- | --- |
|  |  | MAP | HDI 95% | MAP | HDI 95% | MAP | HDI 95% |
| *Non-beneficial synonymous fitness effect* | $w_{s}$ | 0.935 | (0.451, 1) | 0.946 | (0.482, 1) | 0.897 | (0.651, 1) |
| *Non-beneficial nonsynonymous fitness effect* | $w_{ns}$ | 0.847 | (0.259, 0.98) | 0.797 | (0.256, 0.99) | 0.679 | (0.311, 0.922) |
| *Beneficial fitness effect* | $w_{b}$ | 2.012 | (1.334, 2.887) | 1.769 | (1.191, 2.582) | 1.822 | (1.508, 2.246) |
| *Beneficial synonymous* SNV *probability* | $p_{bs}$ | 0.0026 | (0, 0.006) | 0.001 | (0.001, 0.005) | 0.0008 | (0, 0.0022) |
| *beneficial nonsynonymous* SNV *probability* | $p_{bns}$ | 0.0072 | (0.003, 0.01) | 0.006 | (0.006, 0.01) | 0.0073 | (0.0032, 0.01) |
| *average number of synonymous* SNVs *per genotype in the initial population* | $M_{s}$ | 0.539 | (0.455, 0.6) | 0.545 | (0.545, 0.6) | 0.533 | (0.425, 0.6) |
| *average number of nonsynonymous* SNVs *per genotype in the initial population* | $M_{ns}$ | 0.773 | (0.705, 0.874) | 0.781 | (0.781, 0.879) | 0.799 | (0.711, 0.887) |
| *Initial log-fitness correlation* | $\delta$ | 0.0499 | (0, 1.361) | 0.03 | (0, 1.242) | 0.0698 | (0,1.112) |
| *Epistasis* | $\eta$ | -0.1 | (-1, 1221) | 0.296 | (-1, 1.395) | -0.317 | (-1, 0.443) |

**Supplementary Table 3.** Average number of synonymous or non-synonymous mutations per genome across founding populations. Shown are three founding populations (p0) and a single passage 1 population, with the latter representing the experiment described herein. Each such population is based on the selection of one plaque and overnight growth incubation of the phage from the plaque. Following this, short-read deep sequencing was performed as described in (Meir, et al. 2020). The average number of mutations per genome was inferred based on the sum of minor allele frequencies across the genome. The values in this table were used to broadly determine the prior distributions of $M_{s}$ and $M_{ns}$ (Methods).

|  | **synonymous** | **nonsynonymous** |
| --- | --- | --- |
| **p0 (1)** | 0.46 | 0.79 |
| **p0 (2)** | 0.40 | 0.80 |
| **p0 (3)** | 0.40 | 0.82 |
| **p1** | 0.41 | 0.79 |


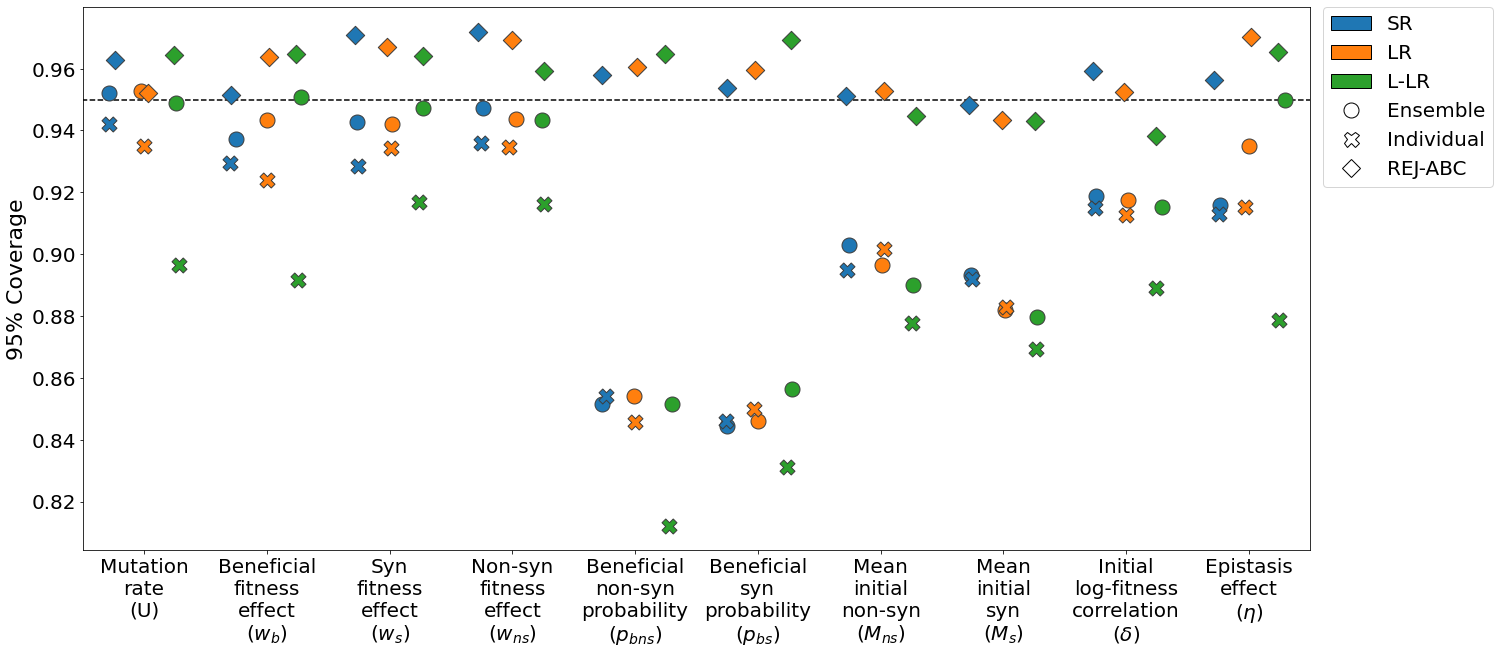


**Supplementary Figure S1. 95% Coverage property of different inference methods on synthetic data.** The coverage property tests that the inferred 95% credible interval contains the true parameter in 95% of the cases. Ensemble SNPE (eight estimators, each trained on 10,000 training examples) has better coverage than individual SNPE (trained on the entire 80,000 training dataset). REJ-ABC almost always has higher coverage, a result of much wider confidence intervals; REJ-ABC is also less accurate (Fig. 3). SR, LR, and L-LR stand for short-reads, long-reads and labeled long-reads summary statistic, respectively.


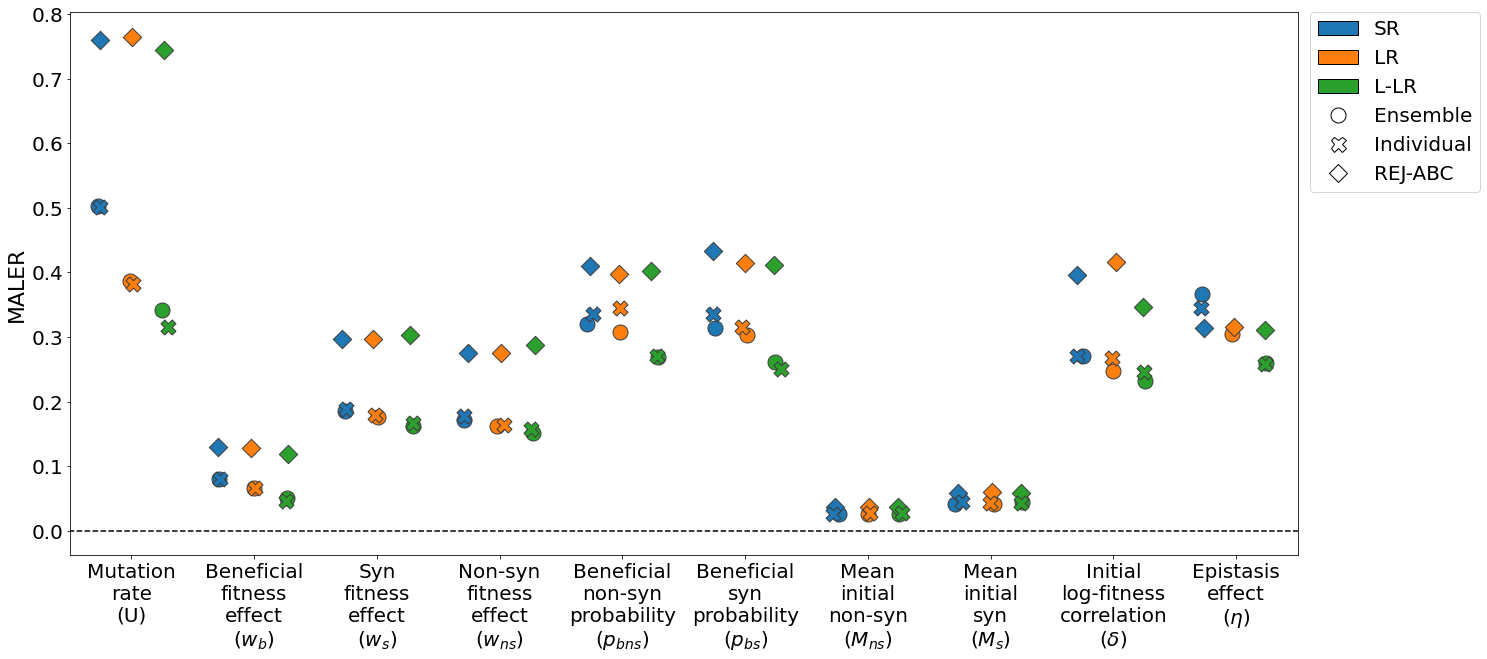


**Supplementary Figure S2. Estimation accuracy for different inference methods on synthetic data.** The mean absolute log error ratio of the maximum a-posteriori (MAP) estimates and the true value. Lower is better, zero is best. Evaluation done on a synthetic dataset of 2,000 simulations that were simulated by using parameters sampled from the same prior as the training dataset. SR, LR, and L-LR stand for short-reads, long-reads and labeled long-reads summary statistic, respectively.

###
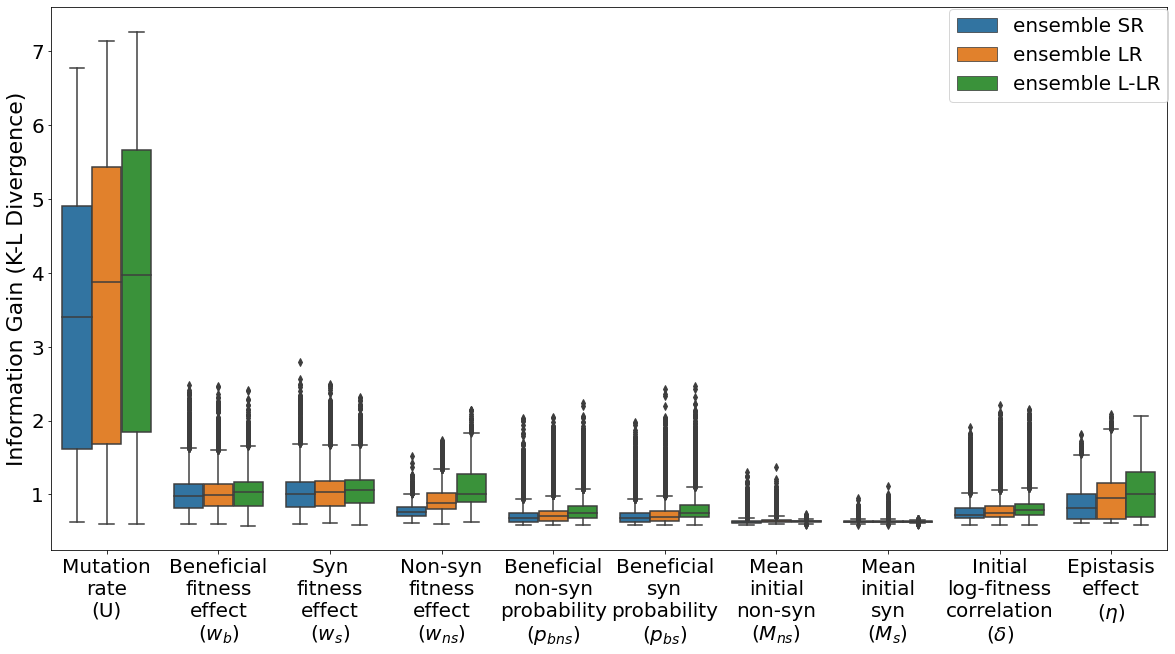


**Supplementary Figure S3. Information Gain for different summary statistics.** The information gain is the Kullback-Leibler (KL) divergence between posterior and prior distributions. Higher is better. Evaluation done on a synthetic dataset of 2,000 simulations that were simulated by using parameters sampled from the same prior as the training dataset. SR, LR, and L-LR stand for short-reads, long-reads and labeled long-reads summary statistic, respectively.


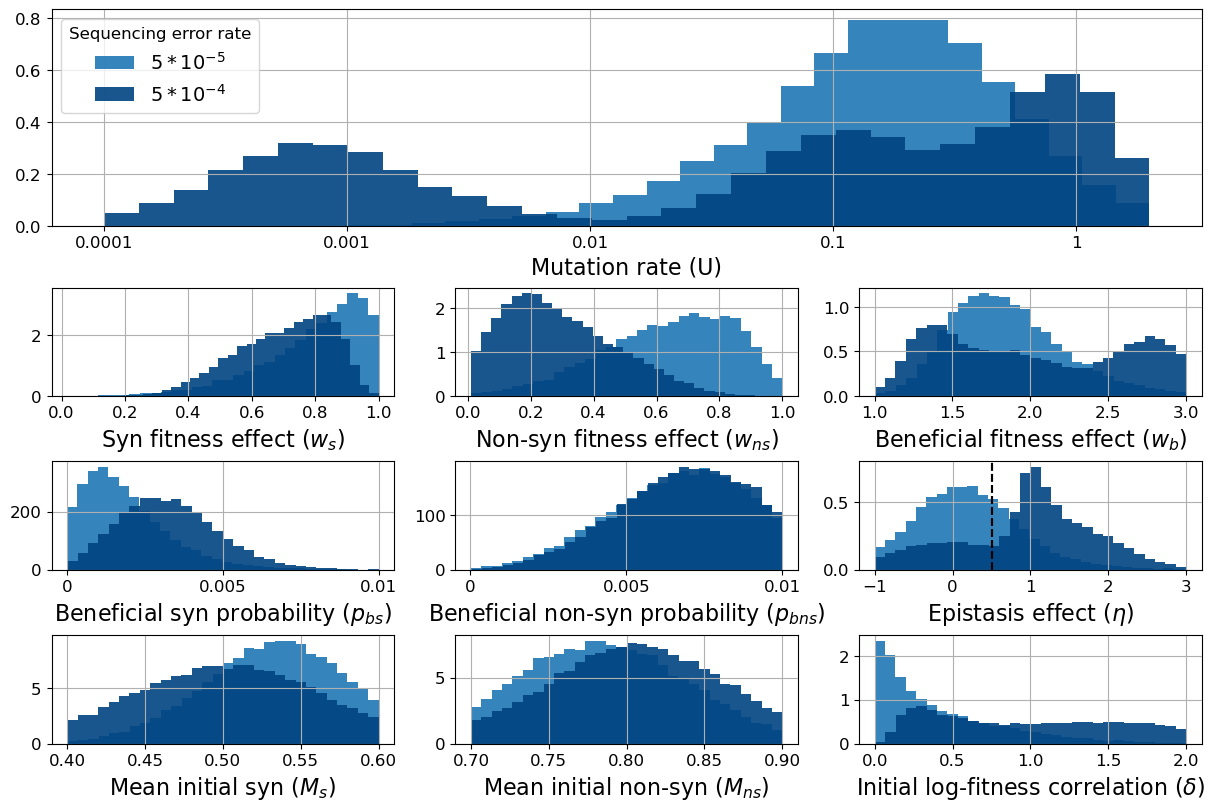


**Supplementary Figure S4. Effect of sequencing error on parameter estimation: LR summary statistic.** Marginal posteriors of all model parameters compared with tenfold higher sequencing error rate. Inferences are from SNPE with long-reads (LR) summary statistic.


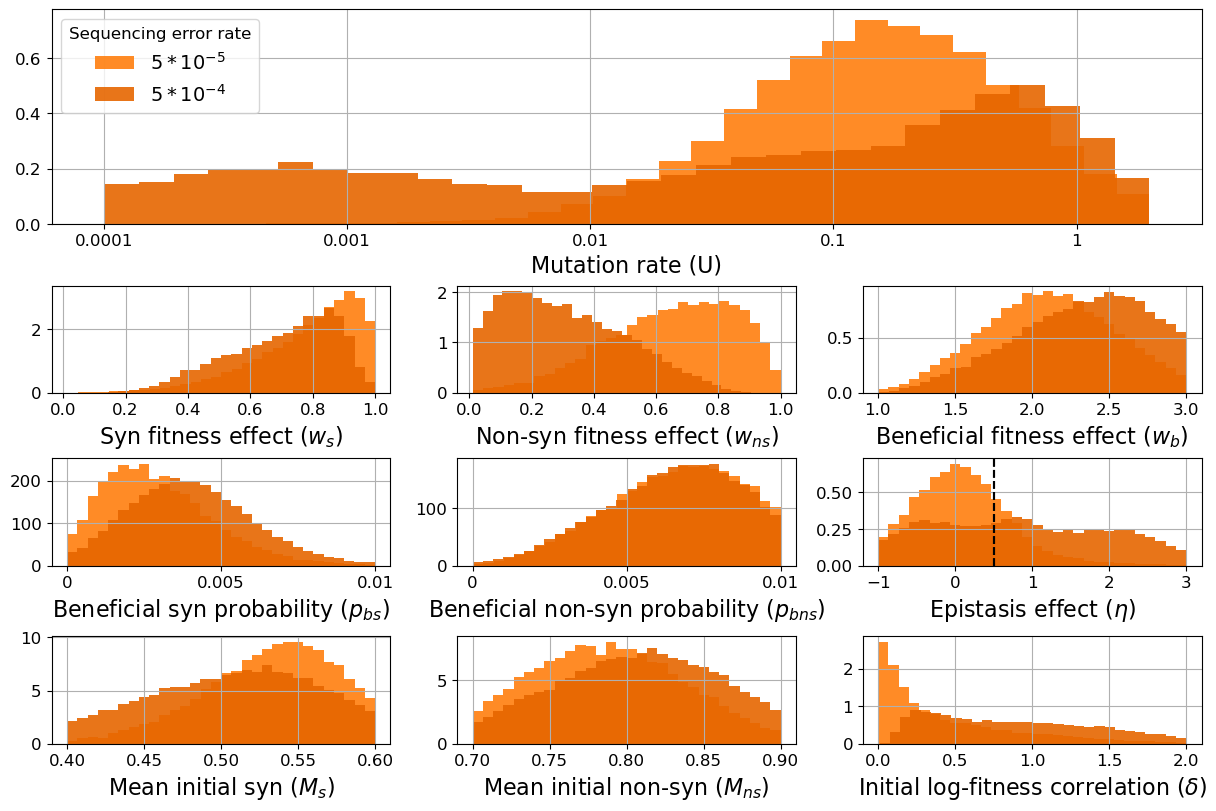


**Supplementary Figure S5. Effect of sequencing error on parameter estimation: SR summary statistic.** Marginal posteriors of all model parameters compared with tenfold higher sequencing error rate. Inferences are from SNPE with short-reads summary statistic (SR).


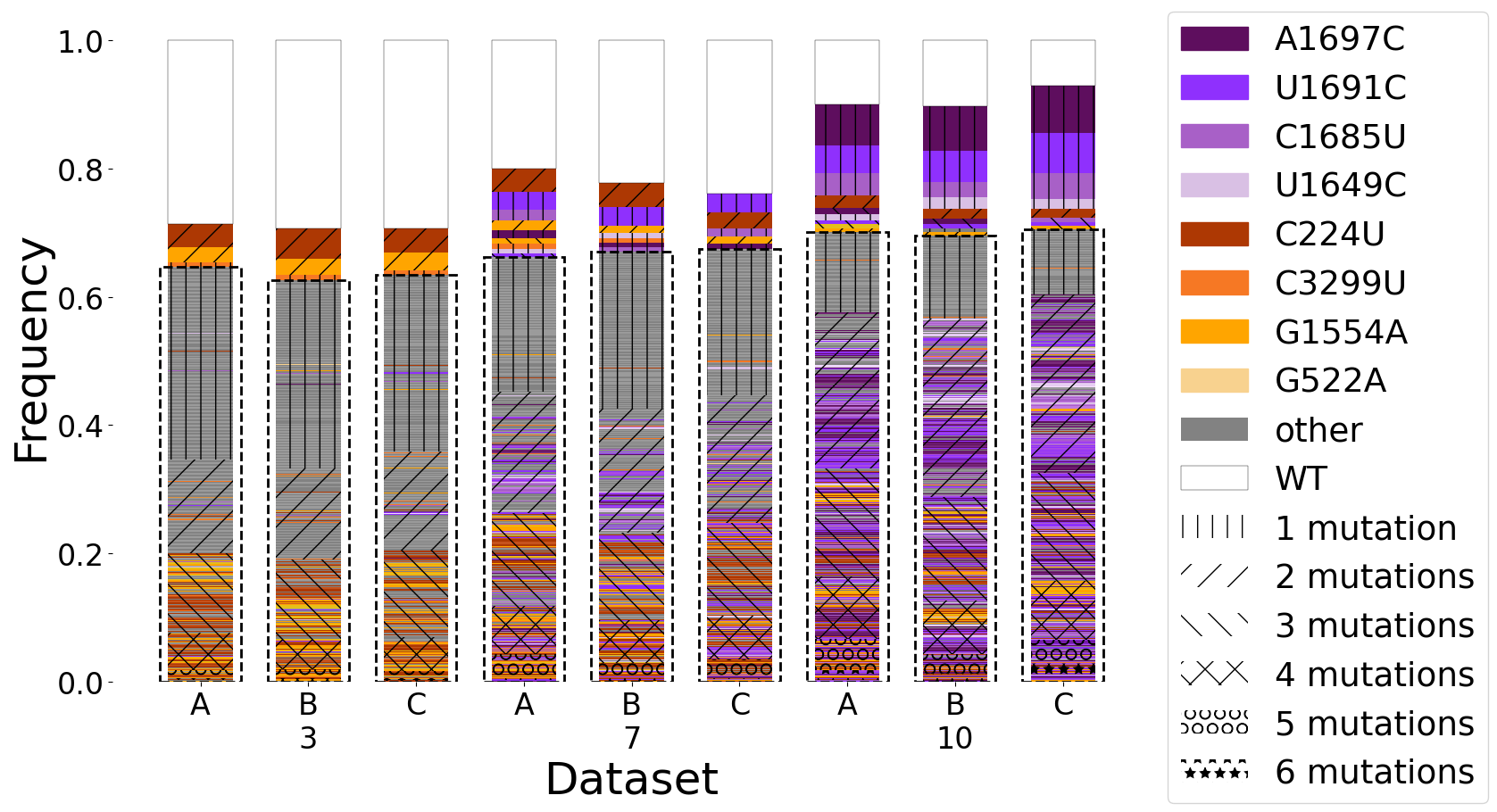


**Supplementary Figure S6.** **Genetic Diversity.** For each replica and passage we show the different genotypes color coded by the SNVs they bear. When a genotype contains more than one high frequency SNV its color is defined by the first one in order of appearance in the legend. The markings on the bars represent the number of SNVs per genotype. The genotypes are ordered by their frequency in each dataset and the number of mutations within them. Rare genotypes are defined as present in less than 0.5% of the sample and are boxed with a dashed line.


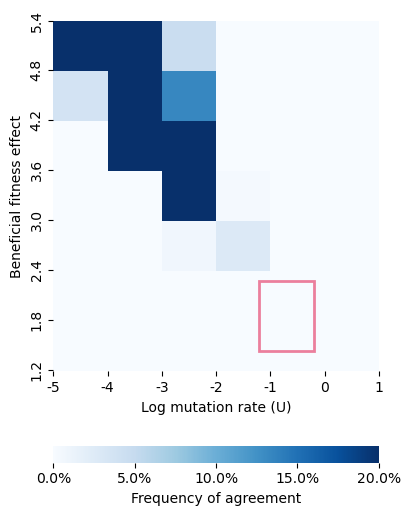


**Supplementary Figure S7.** **Testing whether mutation and selection alone allow negative linkage disequilibrium.** We simulated 12,600,000 instances of a Wright-Fisher two-locus bi-allelic model with selection, mutation, and drift with mutation rate $U$ sampled uniformly from $(5\cdot10^{-5},10)$, fitness effects sampled uniformly from (1.2, 5.4), and effective population size of 2·10^7^ for 10 generations. For each simulation we determined if, at passage 10, the double mutant frequency was lower than or equal to the empirical frequency (Fig. 2) and if the single mutant frequency was higher than or equal to the empirical frequency. The figure shows the frequency of such agreements between simulations and empirical data. Such agreements are likely for extremely low mutation rates (dark blue), which are uncharacteristic for viruses, and unrealistically high fitness effect values. The red box marks the boundaries of the HDI 95% posterior distribution from our main analysis for U and $w_{b}$, and 0% agreement was found in this scenario.


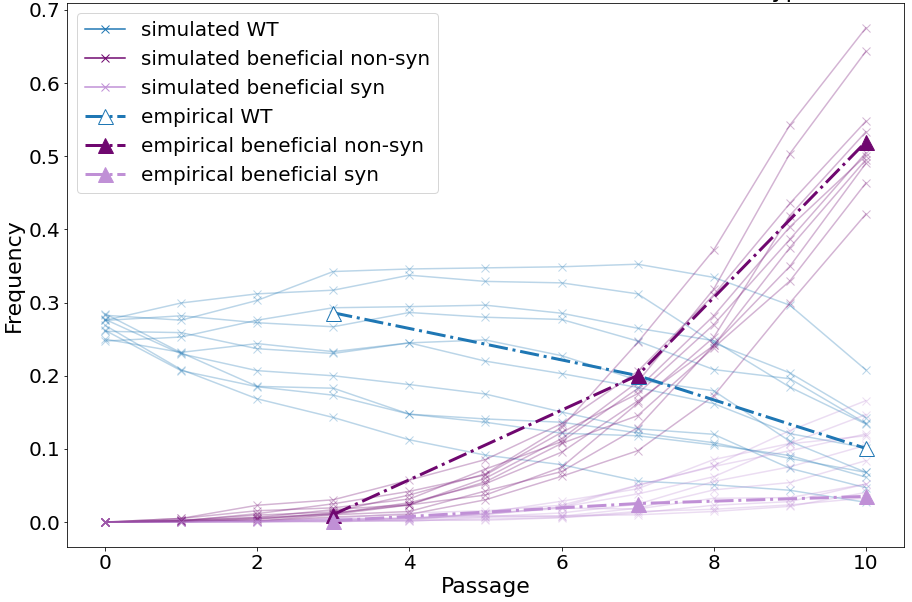


**Figure S8. Posterior predictive check of wildtype and beneficial SNVs frequency dynamics.** The figure compares 10 posterior predictions of the frequency dynamics (solid lines) to the empirical data of population A (dashed bold lines). Predictions were generated by simulating the evolutionary model with parameter sets sampled from the posterior distribution inferred with ensemble SNPE with the L-LR summary statistic.


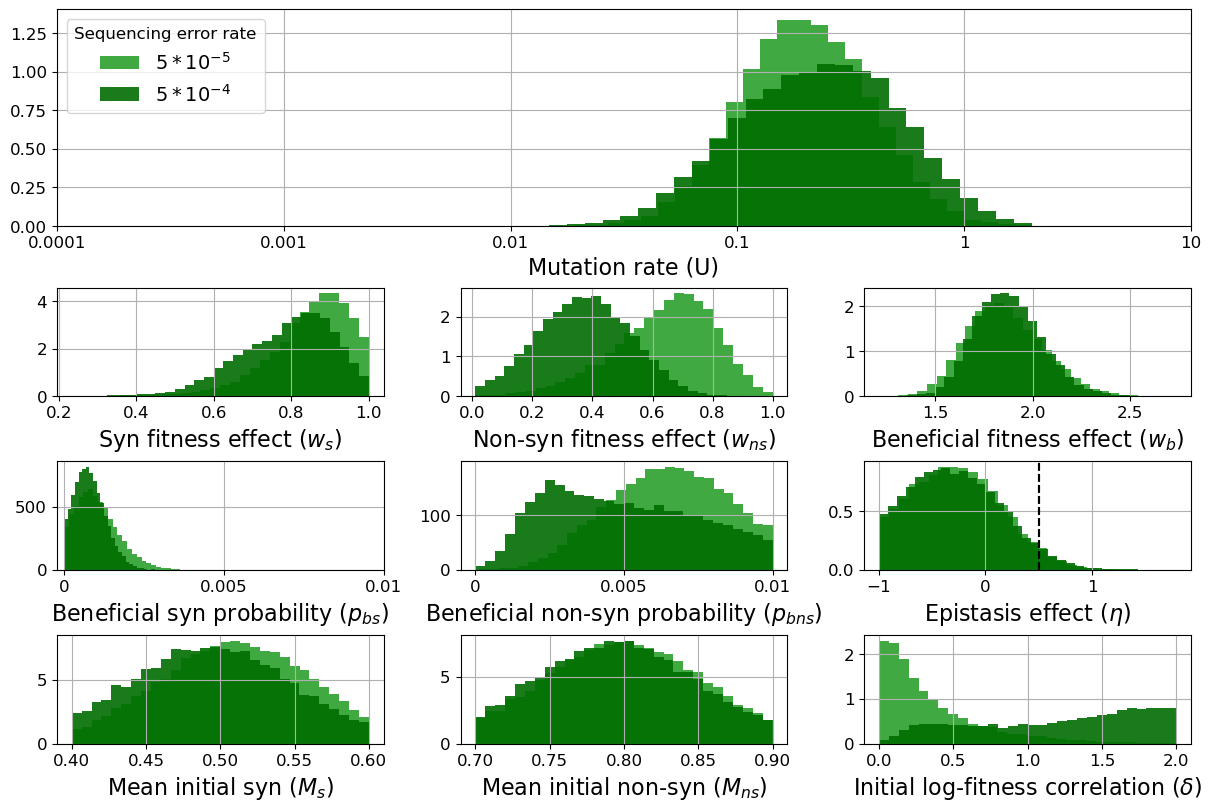


**Supplementary Figure S9. Effect of sequencing error on parameter estimation: L-LR summary statistic.** Marginal posteriors of all model parameters compared with tenfold higher sequencing error rate. Inferences are from SNPE with labeled long-reads summary statistic (L-LR).


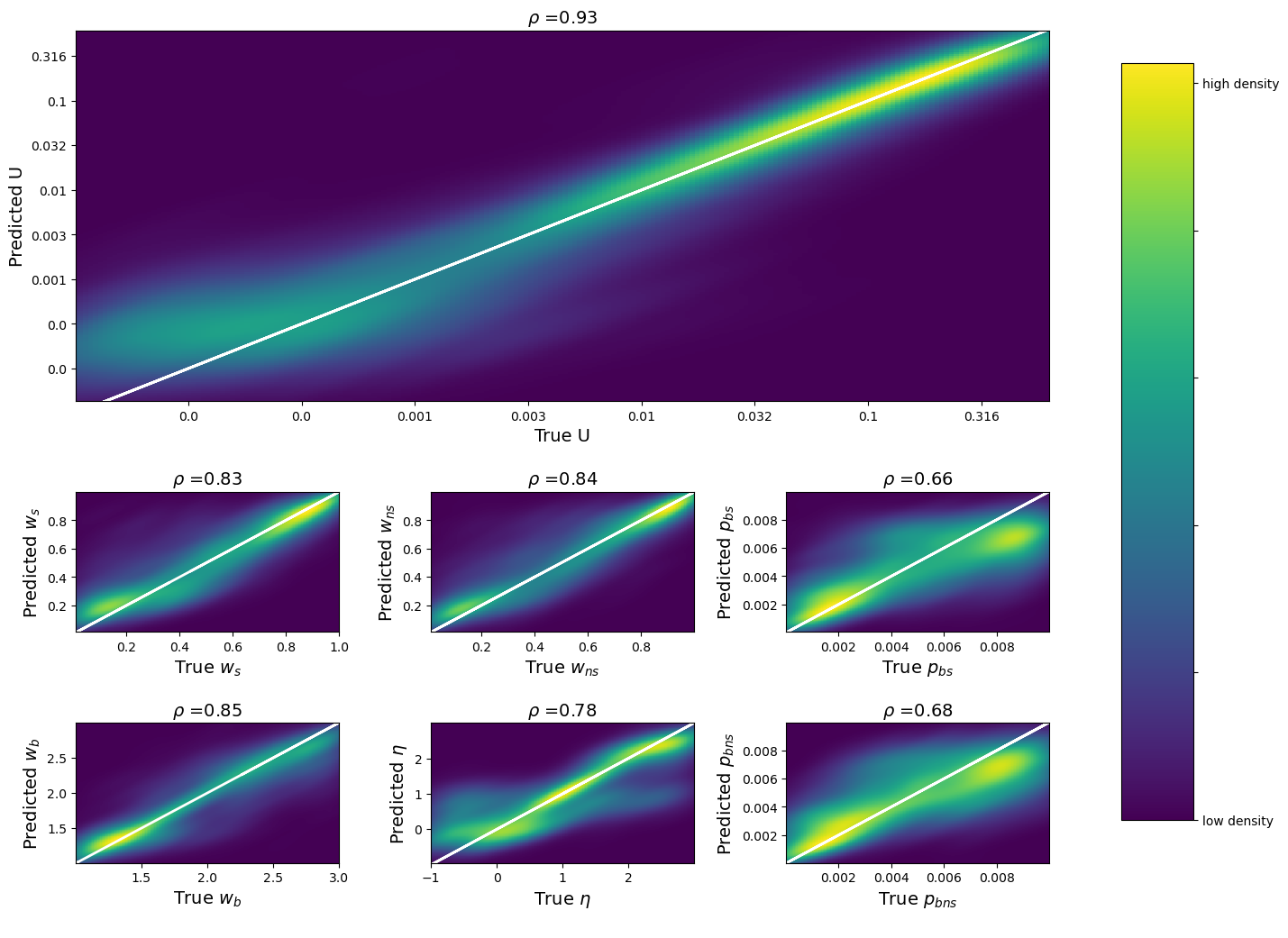


**Supplementary Figure S10. True vs. estimated parameter values on synthetic data using ensemble SNPE and L-LR summary statistic.** The figure shows the true parameter value (x-axis) and the maximum a-posteriori (MAP; y-axis) from 2,000 synthetic datasets. The white diagonal line represents a perfect estimate (y=x). We show kernel density estimates of 2,000 points, where yellow is high density and purple is low density, and the Pearson correlation ρ is computed on these 2,000 points.


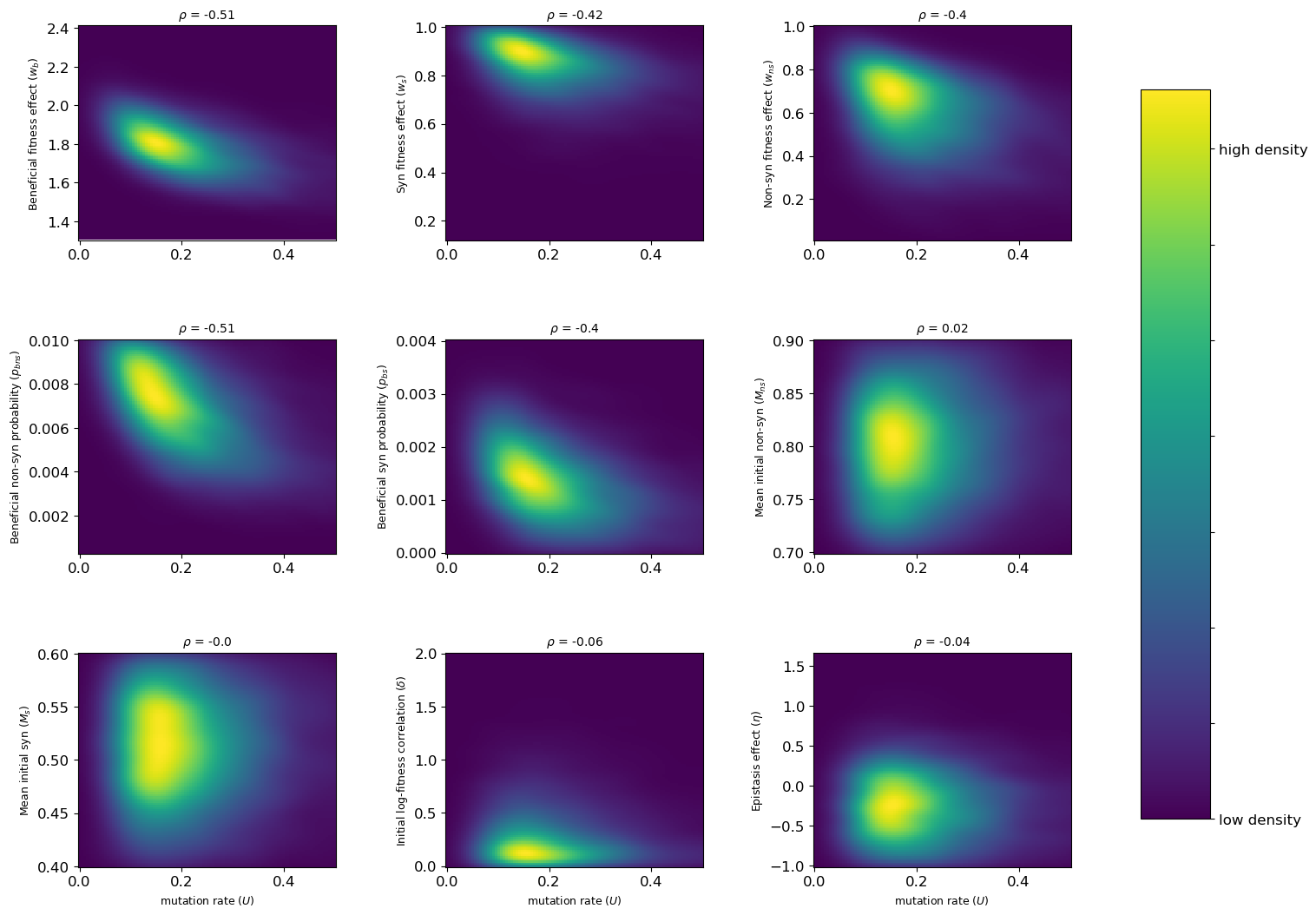


**Supplementary Figure S11. Joint posterior distributions inferred from MS2 empirical data using ensemble SNPE and L-LR summary statistic.** The figure shows the joint posterior distribution of the mutation rate (U; x-axis) and other model parameters (y-axis) estimated on the MS2 empirical data. ρ stands for Pearson correlation of the posterior samples used to produce the plot. The mutation rate is negatively correlated with fitness effects and beneficial probabilities, as can be expected when the two forces are acting together.


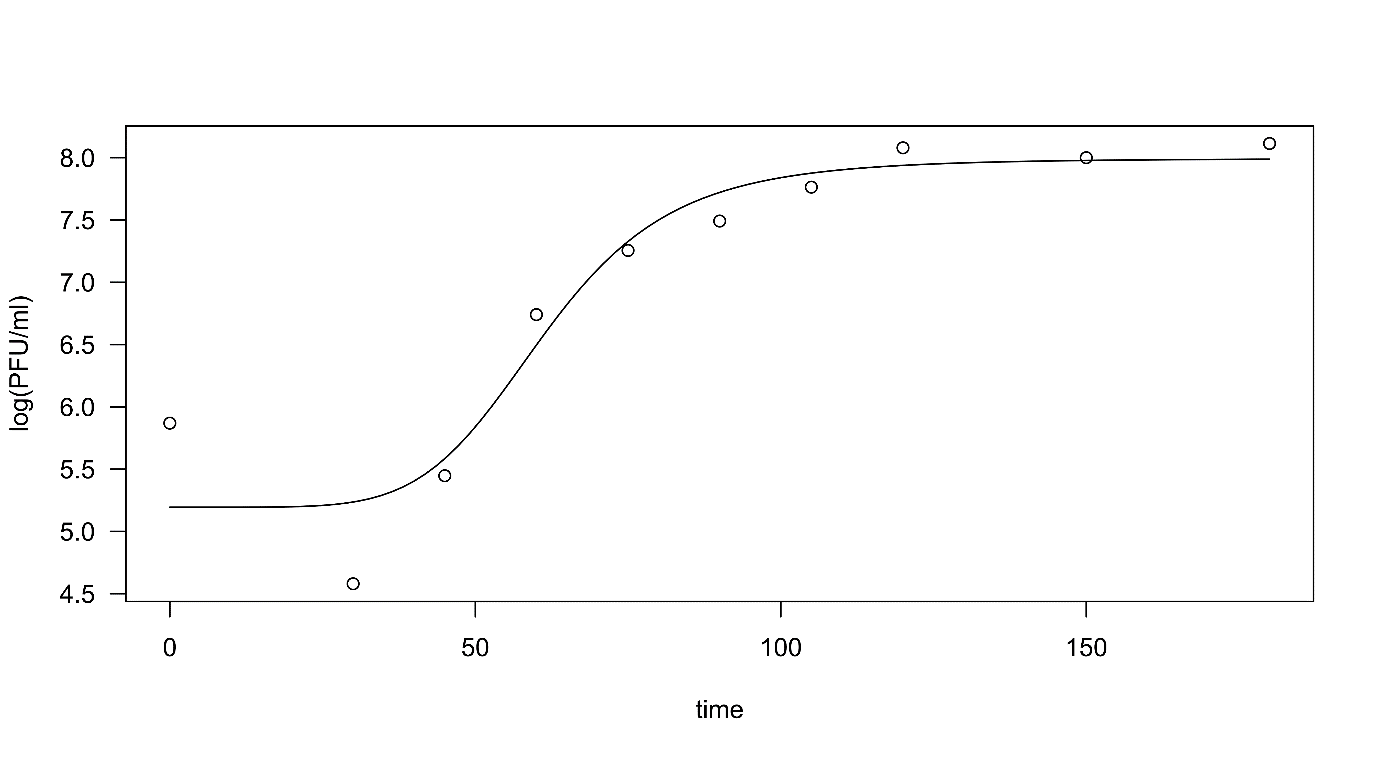
**Supplementary Figure S12**. **One-step growth curve of MS2.** A culture of *E. coli* c-3000 was grown to OD_600_=0.5 and infected with MS2 at MOI=0.1. Phages were collected at the indicated time points and phage concentration was determined by plaque assay.


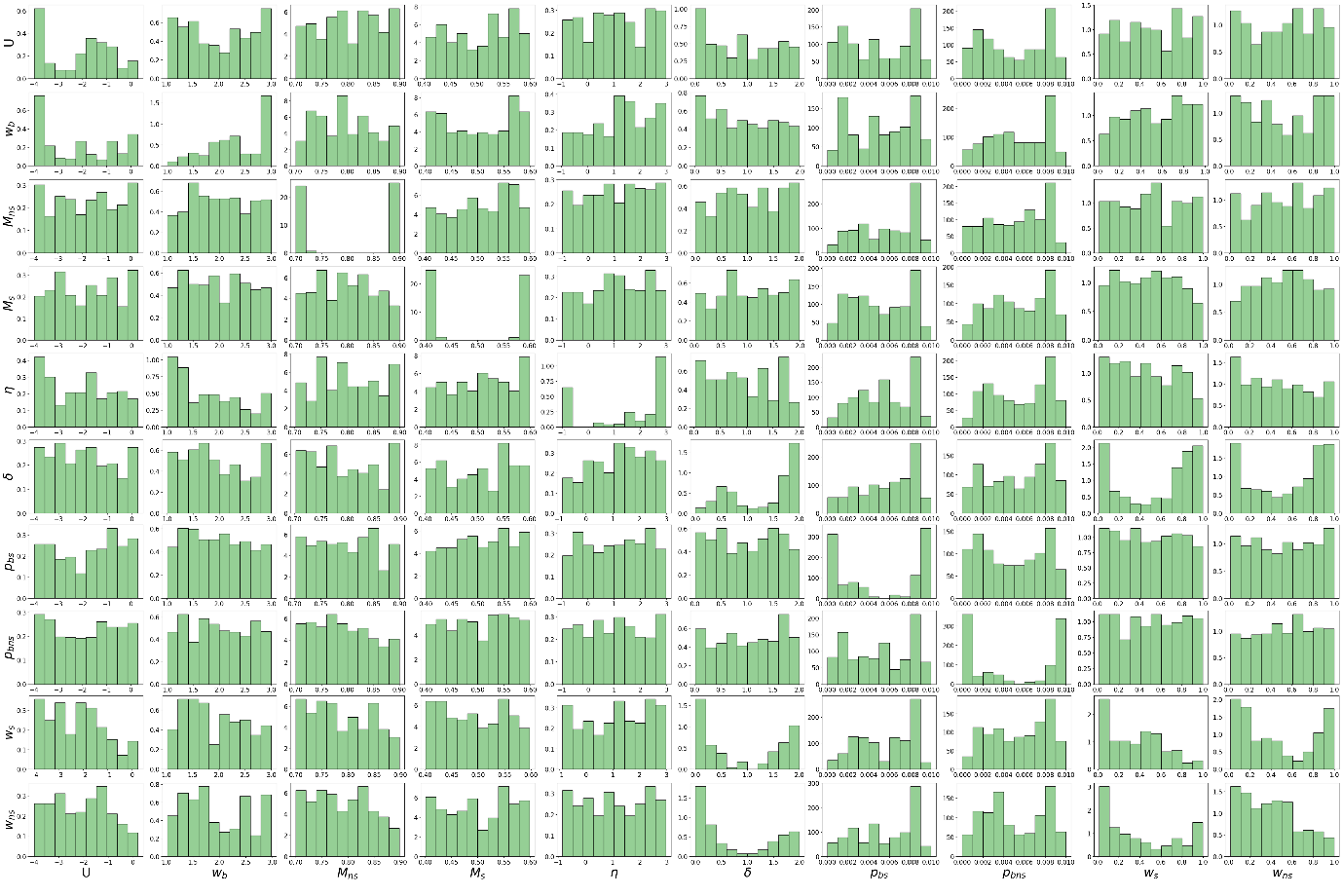


**Supplementary Figure S13. Model parameter values that lead to estimation errors.** We analyzed the inference results of ensemble SNPE with L-LR summary statistic on 2,000 synthetic datasets. For every model parameter (row), we identified datasets in which the true parameter value is outside of the inferred 95% HDI. In these datasets, we examined the histograms of true parameter values (columns). Overall, most histograms appear uniform. In some cases, extreme true values are common, suggesting that such values can lead to inference errors.


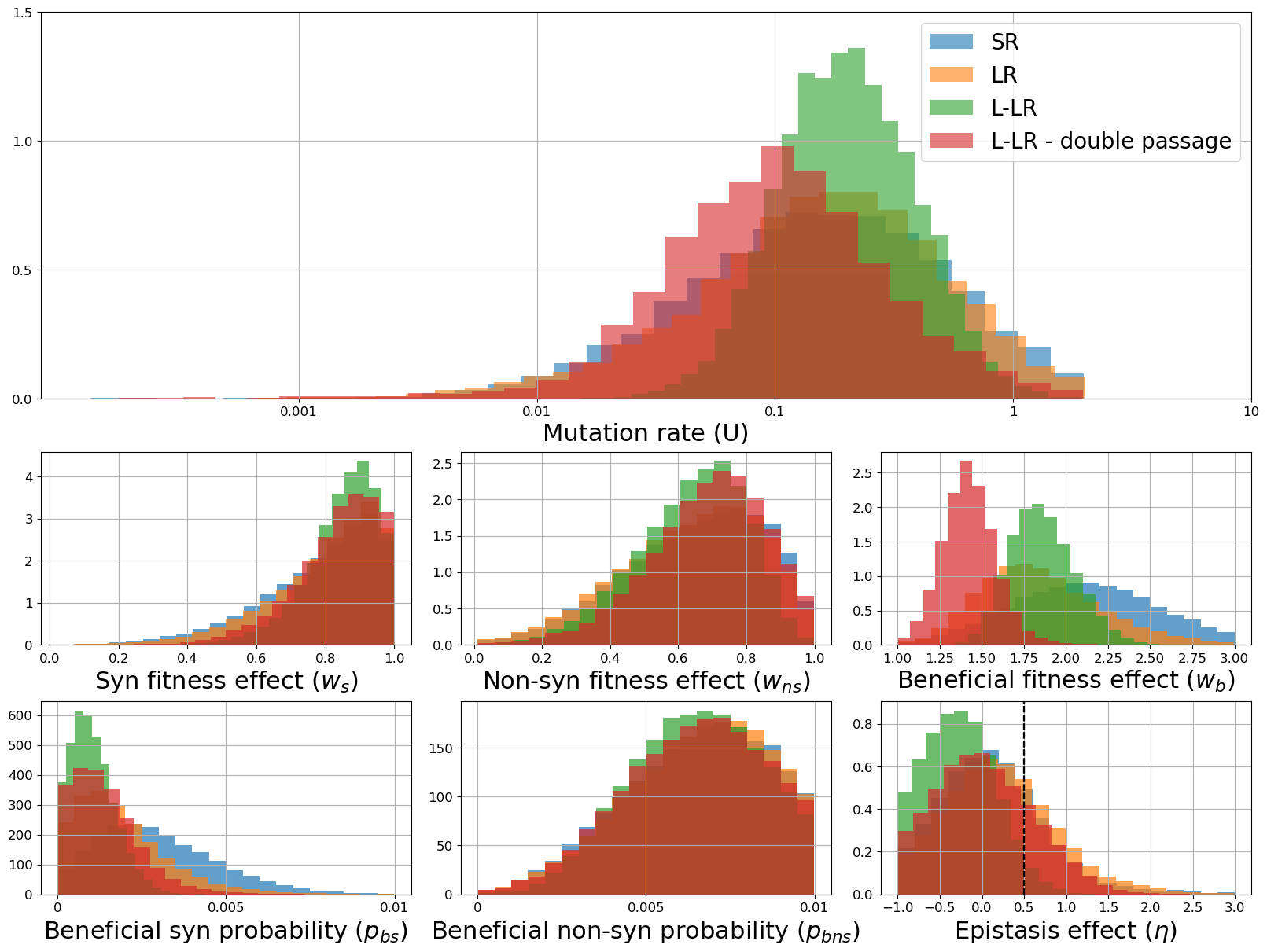


**Supplementary Figure S14. Posterior distributions of model parameters inferred for MS2 under two replication cycles per passage.** Posterior distributions of the model parameters (as in Fig. 4) assuming one replication cycle per passage (blue, orange, and green for SR, LR, and L-LR summary statistic) and assuming two replication cycles per passage (red for L-LR). Shown are marginal posteriors of model parameters using ensemble SNPE. The MAP estimates for the mutation rate *U* with one and two replication cycles per passage are 0.194 (0.056, 0.73 95% HDI) and 0.064 (0.006, 0.618) mutations per genome per replication cycle, respectively, roughly a three-fold change.
